# Comparison of magnetic resonance imaging findings in 880 temporomandibular disorder patients of different age groups: a retrospective study

**DOI:** 10.1186/s12903-022-02666-5

**Published:** 2022-12-28

**Authors:** Chuanjie Li, Qingbin Zhang

**Affiliations:** Department of Temporomandibular Joint Surgery, Affiliated Stomatology Hospital of Guangzhou Medical University, Guangdong Engineering Research Center of Oral Restoration and Reconstruction, Guangzhou Key Laboratory of Basic and Applied Research of Oral Regenerative Medicine, Guangzhou, 510000 China

**Keywords:** TMD, Magnetic resonance imaging, Joint effusion, Disc displacement, Joint position, Disc shape

## Abstract

**Background:**

Magnetic resonance imaging (MRI) findings of temporomandibular joint (TMJ) in temporomandibular disorder (TMD) patients of different ages are still unclear. The aim of this study was to analyze and compare the characteristics of MRI features of TMJs in different age groups.

**Methods:**

A total of 1760 TMJs from 880 patients were included in the study and divided into three groups: ≤ 18Y (n = 195, 14.89 ± 2.35Y); 19-30Y (n = 475, 24.09 ± 3.23Y); and > 30Y (n = 210, 41.73 ± 10.45Y). T2-weighted image (T2WI) of MRI was obtained to evaluate the relationship between age and disc morphology, the degree of disc displacement, joint effusion, joint movement and changes of condylar bone morphology. Data were analyzed by Pearson Chi square test and Spearman correlation coefficient.

**Results:**

There was no statistical difference between left and right sides in all age groups. Except condylar morphology (χ2 = 0.943, P = 0.624), there were significant differences in the distribution of disc morphology, disc position, joint effusion and joint motion among different age groups (χ2 = 24.450, χ2 = 24.829, χ2 = 19.855, χ2 = 39.259, respectively). There were significant differences in the distribution of the degree of anterior disc displacement, condyle morphology and joint effusion in different types of disc morphology among the different age groups (except for joint effusion in > 30Y), among which the first two were significantly correlated with the disc morphology.

**Conclusions:**

The morphology and position of the articular disc changed significantly with age, but the proportion of abnormal condylar bone remained about 50%. The greater the degree of disc folding, the more prone to bone abnormalities.

*Trial registration* This study was retrospectively registered on 28/03/2022 and endorsed by the Ethics committee (LCYJ2022014).

## Background

Temporomandibular disorder (TMD) is a general term for a series of diseases including masticatory muscle, temporomandibular joint (TMJ) and surrounding structures that affect up to about 40% of the population [1]. Pain, joint noises and limited mouth opening are the main reasons for patients seeking medical attention. According to the Diagnostic Criteria for Temporomandibular Disorders (DC/TMD) in 2014 [2], TMD comprises two main groups of disorders, namely painful disease (myalgia, arthralgia and headache attributed to TMD) and TMJ disease (disc displacement disorders, degenerative joint disease, and subluxation). TMJ internal derangement (TMJ ID) is a disorder involving disc and condyle, and up to 80% of TMD patients are accompanied by TMJ ID symptoms [3].

The diagnosis of TMD requires both clinical examination and imaging evaluation around the TMJ area. Magnetic resonance imaging (MRI), computed tomography (CT), cone beam CT, TMJ arthrography, panoramic radiography, plain radiography, transcranial radiography can be used to image the TMJ [4]. Among them, MRI is considered to be the most reliable non-invasive way to evaluate TMD ID due to its advantages in soft tissue imaging [5]. T1 weighted images(T1WI), T2 weighted images (T2WI) and proton-density images (PDWI) are required and the most frequent used section thickness is 3 mm. Disc shape and position, synovial membrane, lateral pterygoid muscle can be clearly displayed by MRI, as well as the early signs of TMJ dysfunction, like thickening of anterior or posterior band, rupture of retrodiscal tissue, changes in disc shape, joint effusion [6]. MRI can also reflect the changes of condylar bone to some extent, although it is not as good as CBCT.

Previous studies focused mainly on the correlation of clinical examination with MRI in TMD. Boboc et al. [7] found that adolescents with GJH have a greater risk of developing TMJ disc displacement, especially disc displacement without reduction. Emshoff et al. [8] analyzed the correlation between TMJ pain and MRI findings, and reported that the MRI findings of osteoarthritis, joint effusion, and bone marrow edema were correlated with TMJ pain. And, Hosgor et al. [9] reported joint effusion increased in direct proportion to the severity of pain and disc displacement. In terms of gender, Luo et al. [10] investigated the size and morphology of articular disc and condyle in young asymptomatic adults and found the thickness of the anterior band of the disc and the media-lateral dimensions of the condylar head were gender-related. On the contrary, Demir et al. [11] compared TMD patients' complaints, clinician's examination findings, and MRI findings by considering gender and found no significant difference. MRI findings of different ages are still unclear.

Unlike other joints (the disease worsens with age), TMD is a common disease with a peak incidence at childbearing age [12]. The MRI images based on oblique sagittal adiposity-suppressed T2W1 in TMD patients in different age groups were retrospectively analyzed and compared, including disc morphology, disc position, joint effusion, condyle movement and condyle morphology. The objective of this study is to provide reference for clinical diagnosis and treatment.

## Methods

### Participants

This study was approved by the Ethics Committee of the Hospital (Approval number: LCYJ2022014) and conformed to the Declaration of Helsinki. Patients diagnosed with TMD according to DC/TMD and underwent MRI examination in the TMJ Diagnosis and Treatment Center from October 2021 to October 2022 were included in the study. Informed consent to participate was obtained from all the participants and from legally authorized representatives of minors age below 16Y. A total of 1760 TMJs from 880 patients were divided into three groups: ≤ 18Y (n = 195); 19-30Y (n = 475); and > 30Y (n = 210).

### Inclusion and exclusion criteria

All patients were received clinical examination according to DC/TMD, and diagnosed with TMD. MRI imaging were obtained before treatment.

*Inclusion criteria* (1) sufficient valid clinical data exist for statistical analysis; (2) agree to participate in the study.

*Exclusion criteria* (1) insufficient valid clinical data exist for statistical analysis; (2) contraindications to MRI imaging; (3) MRI images are of poor quality and cannot be accurately evaluated; (4) maxillofacial trauma or TMJ surgery history.

### Magnetic resonance imaging

All patients underwent MRI imaging of bilateral TMJs with closed and open mouth position using a 3.0 T MRI system. T1WI, T2WI and PDWI sequences of oblique sagittal and coronal images were obtained. Section thickness is set to 3 mm. MRI images based on oblique sagittal adiposity-suppressed T2W1 were evaluated.

The patient was supine with the midpoint of the connection between the two external auditory canals in the center. The median sagittal plane of the face is perpendicular to the ground plane, while the orbital ear plane is perpendicular to the ground plane. The center of the coil is aligned and close to the TMJ area. Patients wear earplugs to protect their hearing. The patients are instructed to maintain the intercuspal position for closed mouth MRI imaging, and to open the mouth as much as possible and bite the mouth opener.

### Evaluation indicators

#### Disc shape

**Fig. 1:**
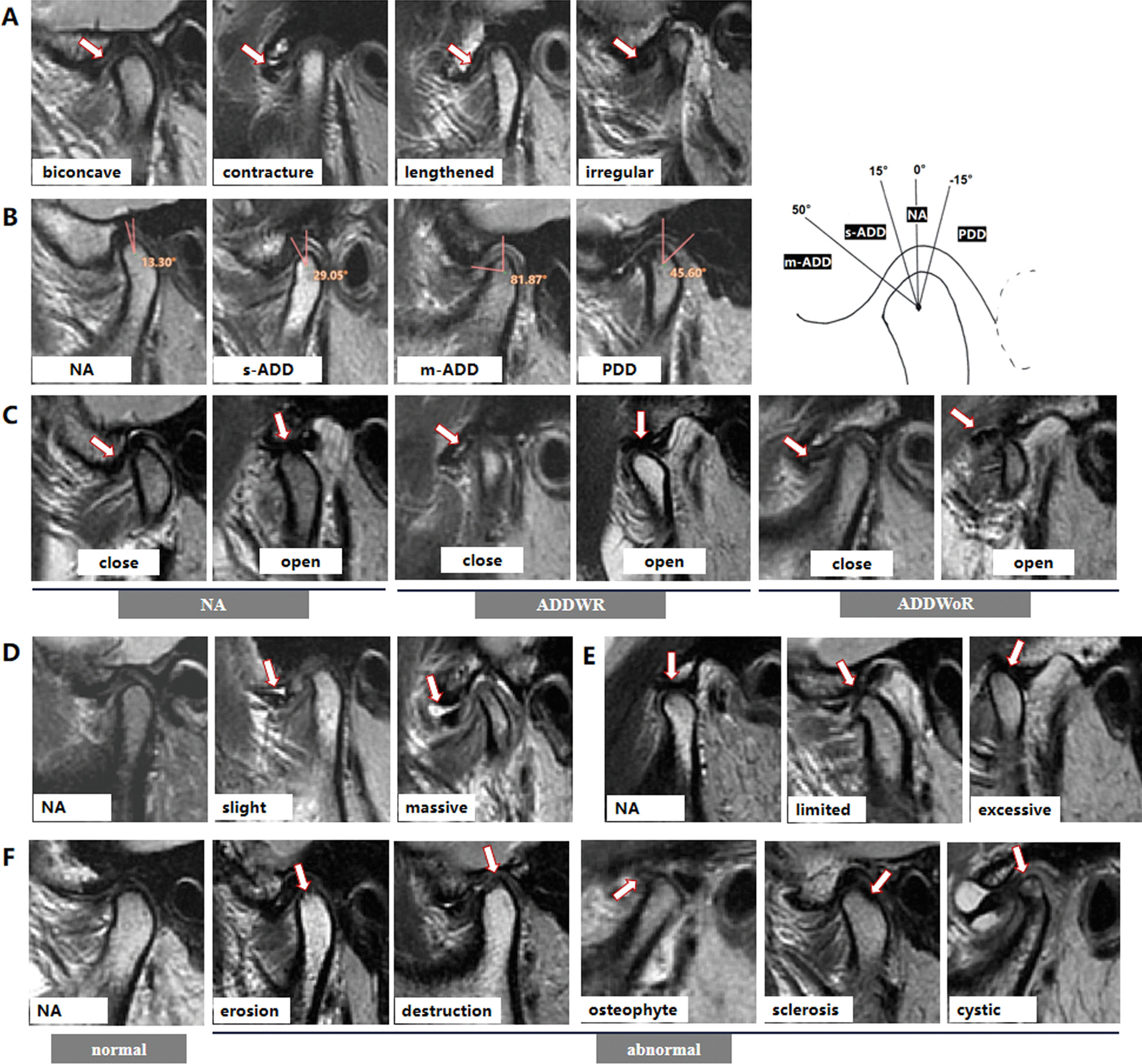
MRI imaging. **A** The shape of disc (the arrow indicates the articular disk); **B** the position of disc (the Angle between the posterior band and the condylar head in the closed-mouth position); **C** classification of anterior articular disc displacement (depending on the position of the articular disc in the opening position); **D** joint effusion (the arrow indicates the effusion); **E** joint motion (the position of the condyle with respect to the articular tubercle in the opening position); **F** condylar bone morphology (the arrow indicates the bone abnormality). Where: NA = normal; s-ADD = slight to mild anterior disc displacement; m-ADD = moderate to severe anterior disc displacement; PDD = posterior disc displacement; ADDWR = anterior disc displacement with reduction; ADDWoR = anterior disc displacement without reduction

The disc morphology was evaluated at closed-mouth position. The normal articular disc has a double concave disc-like black band shadow, with clear edges and clear boundaries with surrounding tissues. In this study, as showed in Fig. 1A, the disc shape was divided into the following four types which incomplete reference to Orhan's classification[13]: (1) biconcave: Narrowed intermediate zone and fully visible posterior and anterior bands; (2) contracture; (3) lengthened: Equal thickness of the anterior, intermediate, and posterior band; (4) irregular: Irregular strip or nodules disc shape, or the disc is missing in shape and the signal is discontinuous.

#### Disc position

The disc position was evaluated at closed-mouth position. The normal disc position is the posterior band located directly superior to the condylar head in the closed-mouth position[14]. There is a clear boundary between the posterior band of articular disc and the double plate area, and the Angle between the posterior band and the vertical line of the condyle at 12 points is the disc boundary Angle[15]. As showed in Fig. 1B, 4 types were evaluated: (1) −15 to 15° indicates the normal position; (2) 15–50° indicates slight to mild anterior disc displacement (s-ADD); (3) > 50° indicates moderate to severe anterior disc displacement (m-ADD); (4) < −15° indicates the posterior band positioned posterior to the condyle (posterior disc displacement, PDD) [16].

The reduction of the anterior displaced articular disc was evaluated by open-mouth imaging. As showed in Fig. 1C, 3 types of disc displacement were evaluated: (1) normal (NA); (2) anterior disc displacement with reduction (ADDWR): the posterior band located anterior to the condylar head in the closed-mouth position, but with a normal disc-condyle relationship in the open-mouth position; (3) anterior disc displacement without reduction (ADDWoR): the posterior band positioned anterior to the condyle in both the closed- and open-mouth positions[17].

#### Joint effusion

The joint effusion was evaluated at closed mouth position. As showed in Fig. 1D, there are 3 levels of fluid accumulation: (1) NA: no high signal image; (2) slight: spot-like or linear high signal shadow; (3) massive effusion: high signal shadow of effusion pool is visible. The classification was based on the presence of high signal shadow in the superior or inferior joint space in T2WI.

#### Joint movement

The joint movement was evaluated at open-mouth position. Based on the relative position of the condyle to the articular tuberosity, they were classified as limited, normal, or excessive as showed in Fig. 1E. (1) limited is defined when at least one joint has limited movement; (2) excessive is defined as hyperactivity when at least one side is hyperactive and there is no limitation of movement; (3) normal is defined when there is no significant limitation or excessive movement on both sides.

#### Condylar bone morphology

The joint movement was evaluated at closed mouth position. As showed in Fig. 1F, it was classified as normal and abnormal according to condyle bone morphology and signal conditions.The abnormalities was defined as the condition in which erosion, destruction, osteophyte, sclerosis or cystic changes was found on the articular surface.

### Data analysis

SPSS Statistics 23.0 software was ued to analyze the eligible data. Age was described by mean ± standard deviation. Chi-Square test was performed for the distribution of gender, joint disk morphology, disk position, joint effusion, condyle movement, and condyle morphology. Pearson Chi-Square test was performed for expected frequency less than 5. The correlations between the study variables were assessed using the Spearman correlation coefficient. *P* < 0.05 was considered statistically significant.

## Results

### Participant characteristics

A total of 880 patients (692 females; 7-76Y) with 1760 TMJs were included in the study and divided into three groups: ≤ 18Y (n = 195, 14.89 ± 2.35Y); 19-30Y (n = 475, 24.09 ± 3.23Y); and > 30Y (n = 210, 41.73 ± 10.45Y). As Table1 showed, the proportion of female in different age groups is significantly larger than that of male (χ2 = 13.187, *P* = 0.001).

**Table 1:** Participant characteristics

Parameter	≤ 18Y (n = 195)	19-30Y (n = 475)	> 30Y (n = 210)	χ2	*P*
Mean age	14.89 ± 2.35Y	24.09 ± 3.23Y	41.73 ± 10.45Y	-	-
Female n(%)	138 (70.8%)	394 (82.9%)	160 (76.2%)	13.187	0.001

The sex ratio was obtained from Pearson Chi-Square test analysis

### MRI features between left and right sides

There was no statistical significance in the composition ratio of disc morphology, disc position, joint effusion, and condyle morphology between the left and right sides of TMJs (Table 2).

**Table 2:** Correlation between the results of MRI in left and right sides

Type	Parameter	Left (n = 880)	Right (n = 880)	*χ2*, P
Disk shape	Biconcave	284 (32.3%)	294 (33.4%)	2.461, 0.482
	Contracture	277 (31.5%)	251 (28.5%)	
	Lengthened	119 (13.5%)	135 (15.3%)	
	Irregular	200 (22.7%)	200 (22.7%)	
Disk position	NA	236 (26.8%)	230 (26.1%)	1.330, 0.722
	s-ADD	70 (8.0%)	83 (9.4%)	
	m-ADD	548 (62.3%)	1087 (61.8%)	
	PDD	26 (3.0%)	54 (3.1%)	
Joint effusion	NA	740 (84.1%)	726 (82.5%)	5.687, 0.060
	Slight	133 (15.1%)	135 (15.3%)	
	Massive	7 (0.8%)	26 (1.5%)	
Condyle	Normal	449 (51.0%)	458 (52.0%)	0.184, 0.703
	Abnormal	431 (49.0%)	422 (48.0%)	

The constituent ratio was obtained from Pearson Chi-Square test analysis. Where: NA = normal; s-ADD = slight to mild anterior disc displacement; m-ADD = moderate to severe anterior disc displacement; PDD = posterior disc displacement. *p* significance was set at < 0.05

### MRI features in different age groups

Pearson Chi-Square test was used to analyze the relationship between different age groups and disc morphology, disc position, joint effusion, condyle movement, and condyle morphology, as showed in Table 3.

**Table 3:** Correlation between the results of MRI in patients and different age groups

Parameter	≤ 18Y (n = 390)	19-30Y (n = 950)	> 30Y (n = 420)	*χ2*, P
*Disk shape*				
Biconcave	105 (26.9%)	320 (33.7%)	153 (36.4%)	24.450, 0.000
Contracture	145 (37.2%)	285 (30.0%)	98 (23.3%)	
Lengthened	56 (14.4%)	143 (15.1%)	55 (13.1%)	
Irregular	84 (21.5%)	202 (21.3%)	114 (27.1%)	
*Disk position*				
NA	93 (23.8%)	246 (25.9%)	127 (30.2%)	24.829, 0.000
s-ADD	17 (4.4%)	95 (10.0%)	41 (9.8%)	
m-ADD	260 (66.7%)	582 (61.3%)	245 (58.3%)	
PDD	20 (5.1%)	27 (2.8%)	7 (1.7%)	
*Joint effusion*				
Na	329 (84.4%)	806 (84.8%)	331 (78.8%)	19.855, 0.001
Slight	58 (14.9%)	136 (14.3%)	74 (17.6%)	
Massive	3 (0.8%)	8 (0.8%)	15 (3.6%)	
*Condyle*				
Normal	199 (51.0%)	499 (52.5%)	209 (49.8%)	0.943, 0.624
Abnormal	191 (49.0%)	451 (47.5%)	211 (50.2%)	
*Joint motion*				
Limited	106 (54.4%)	119 (29.5%)	91 (43.3%)	39.259, 0.000
NA	53 (27.2%)	141 (35.0%)	55 (26.2%)	
Excessive	36 (18.5%)	143 (35.5%)	64 (30.5%)	

The constituent ratio was obtained from Pearson Chi-Square test analysis. Where: NA = normal; s-ADD = slight to mild anterior disc displacement; m-ADD = moderate to severe anterior disc displacement; PDD = posterior disc displacement. *p* significance was set at < 0.05

There were significant differences in the distribution of disc morphology in different age groups (χ2 = 24.450, *P* = 0.000). In ≤ 18Y group, contracture ranked first followed by biconcave, irregular and lengthened. In 19-30Y group, biconcave ranked first followed by contracture, irregular and lengthened. In > 30Y group, biconcave ranked first followed by irregular, contracture and lengthened.

There were significant differences in the distribution of disc position in different age groups (χ2 = 24.829, *P* = 0.000). In ≤ 18Y group, m-ADD ranked first followed by NA, PDD and s-ADD. In 19-30Y group and > 30Y group, m-ADD ranked first followed by NA, s-ADD and PDD.

There were significant differences in the distribution of joint effusion in different age groups (χ2 =  = 19.855, *P* = 0.001). In all three age groups, NA ranked first followed by slight and massive.

There was no significant difference in the distribution of condyle morphology among different age groups (χ2 =  = 0.943, *P* = 0.624).

The distribution of joint motion in different age groups was significantly different (χ2 =  = 39.259, *P* = 0.000). In ≤ 18Y group, limited ranked first followed by NA and excessive. In 19-30Y group, excessive ranked first followed by NA and limited. In > 30Y group, limited ranked first followed by excessive and NA.

### Correlation analysis of MRI evaluation of articular disk morphology at different ages

The proportions of articular disc position, joint effusion and condyle morphology in articular disc morphology were analyzed in different age groups by Pearson Chi-Square test and Spearman correlation coefficient.

There was a statistical difference in the distribution of disc position among different articular disc shapes (χ2 = 1204.523, *P* = 0.000) (Table 4). In biconcave disc, NA ranked the first (60.7%) followed by s-ADD, m-ADD and PDD. In contracture disc, m-ADD ranked the first (96.4%) followed by NA, s-ADD, PDD. In lengthened disc, NA ranked the first (38.6%) followed by m-ADD, s-ADD and PDD. In irregular disc, m-ADD ranked the first (97.0%) followed by NA and s-ADD. Interestingly, in the ≤ 18Y group, 72.4% of the disc position of contracture disc were NA, followed by m-ADD (17.1%), while m-ADD ranked first in the position of lengthened disc. There was a correlation between the degree of anterior disc displacement and the shape of the disc in all age groups (*P* = 0.000). The positive correlation coefficient indicates that disc with more irregular presented more anterior position.

**Table 4:** Correlation between disc morphology and joint position in different age groups

Age group	Position	Biconcave	Contracture	Lengthened	Irregular	*χ2*, P	r, P
≤ 18Y, (n = 390)	NA	76 (72.4%)	1 (0.7%)	15 (26.8%)	1 (1.2%)	333.056, 0.000	0.558, 0.000
	s-ADD	10 (9.5%)	0 (0.0%)	7 (12.5%)	0 (0.0%)		
	m-ADD	18 (17.1%)	144 (99.3%)	18 (32.1%)	80 (95.2%)		
	PDD	1 (1.0%)	0 (0.0%)	16 (28.6%)	3 (3.6%)		–
19-30Y, (n = 950)	NA	184 (57.5%)	1 (0.4%)	60 (42.0%)	1 (0.5%)	647.641, 0.000	0.517, 0.000
	s-ADD	67 (20.9%)	8 (2.8%)	20 (14.0%)	0 (0.0%)		
	m-ADD	65 (20.3%)	275 (96.5%)	41 (28.7%)	201 (99.5%)		
	PDD	4 (1.3%)	1 (0.4%)	22 (15.4%)	0 (0.0%)		–
> 30Y (n = 420)	NA	91 (59.5%)	7 (7.1%)	23 (41.8%)	6 (5.3%)	241.669, 0.000	0.555, 0.000
	s-ADD	34 (22.2%)	1 (1.0%)	6 (10.9%)	0 (0.0%)		
	m-ADD	27 (17.6%)	90 (91.8%)	21 (38.2%)	107 (93.9%)		
	PDD	1 (0.7%)	0 (0.0%)	5 (9.1%)	1 (0.9%)		–
Total (n = 1760)	NA	351 (60.7%)	9 (1.7%)	98 (38.6%)	8 (2.0%)	1204.523, 0.000	0.535, 0.000
	s-ADD	111 (19.2%)	9 (1.7%)	33 (13.0%)	0 (0.0%)		
	m-ADD	110 (19.0%)	509 (96.4%)	80 (31.5%)	388 (97.0%)		
	PDD	6 (1.0%)	1 (0.2%)	43 (16.9%)	4 (1.0%)		–

The constituent ratio was obtained from Pearson Chi-Square test analysis, and the correlation coefficient was obtained from Spearman test. Where: NA = normal; s-ADD = slight to mild anterior disc displacement; m-ADD = moderate to severe anterior disc displacement; PDD = posterior disc displacement. *p* significance was set at < 0.05

In general, there was a statistical difference in the distribution of joint effusion in different disc shapes (χ2 = 32.901, *P* = 0.000) (Table 5), but there was no statistical difference in the > 30Y group (χ2 = 9.817, *P* = 0.133). The incidence of joint effusion in contracture disc was significantly higher than other types. There was no correlation between articular effusion and articular disk morphology in all age groups (*P* > 0.05).

**Table 5:** Correlation between disc morphology and joint effusion in different age groups

Age group	Effusion	Biconcave	Contracture	Lengthened	Irregular	*χ2*, P	r, P
≤ 18Y, (n = 390)	NA	96 (72.4%)	105 (72.4%)	53 (94.6%)	75 (89.3%)	26.469, 0.000	−0.025, 0.623
	Slight	8 (7.6%)	38 (26.2%)	3 (5.4%)	9 (10.7%)		
	Massive	1 (1.0%)	2 (1.4%)	0 (0.0%)	0 (0.0%)		
19-30Y, (n = 950)	NA	282 (88.1%)	224 (78.6%)	124 (86.7%)	176 (87.1%)	13.893, 0.031	0.009, 0.781
	Slight	35 (10.9%)	59 (20.7%)	18 (12.6%)	24 (11.9%)		
	Massive	3 (0.9%)	2 (0.7%)	1 (0.7%)	2 (1.0%)		
> 30Y (n = 420)	NA	129 (84.3%)	73 (74.5%)	40 (72.7%)	89 (78.1%)	9.817, 0.133	0.077, 0.116
	Slight	22 (14.4%)	21 (21.4%)	10 (18.2%)	21 (18,4%)		
	Massive	2 (1.3%)	4 (4.1%)	5 (9.1%)	4 (3.5%)		
Total (n = 1760)	NA	507 (87.7%)	402 (76.1%)	217 (85.4%)	340 (85.0%)	32.901, 0.000	0.023, 0.334
	Slight	65 (11.2%)	118 (22.3%)	31 (12.2%)	54 (13.5%)		
	Massive	6 (1.0%)	8 (1.5%)	6 (2.4%)	6 (1.5%)		

The constituent ratio was obtained from Pearson Chi-Square test analysis, and the correlation coefficient was obtained from Spearman test. Where: NA = normal. *p* significance was set at < 0.05

In general, there was a statistical difference in the distribution of condyle morphology in different disc shapes (χ2 = 420.342, *P* = 0.000) (Table 6). The morphology of articular discs prone to abnormal bone morphology were irregular, contracture, lengthened and biconcave. Interestingly, the proportion of articular bone abnormalities with contracture disc in the > 30Y group was 66.3%, which was significantly higher than that in other age groups. There was a correlation between condyle morphology and disc morphology in all age groups (*P* = 0.000). The positive correlation coefficient indicates that disc with more irregular presented more likely to have bone abnormalities.

**Table 6:** Correlation between disc morphology and joint morphology in different age groups

Age group	Type	Biconcave	Contracture	Llengthened	Iirregular	*χ2*, P	r, P
≤ 18Y, (n = 390)	Normal	87 (82.9%)	65 (44.8%)	34 (60.7%)	13 (15.5%)	89.387, 0.000	0.415, 0.000
	Abnormal	18 (17.1%)	80 (55.2%)	22 (39.3%)	71 (84.5%)		
19-30Y, (n = 950)	Normal	258 (80.6%)	127 (44.6%)	76 (53.1%)	38 (18.8%)	200.669, 0.000	0.427, 0.000
	Abnormal	62 (19.4%)	158 (55.4%)	67 (46.9%)	164 (81.2%)		
> 30Y, (n = 420)	Normal	124 (81.0%)	33 (33.7%)	37 (67.3%)	15 (13.2%)	137.888, 0.000	0.492, 0.000
	Abnormal	29 (19.0%)	65 (66.3%)	18 (32.7%)	99 (86.8%)		
Total, (n = 1760)	Normal	469 (81.1%)	225 (42.6%)	147 (57.9%)	66 (16.5%)	420.342, 0.000	0.440, 0.000
	Abnormal	109 (18.9%)	303 (54.7%)	107 (42.1%)	334 (83.5%)		

The constituent ratio was obtained from Pearson Chi-Square test analysis, and the correlation coefficient was obtained from Spearman test. *p* significance was set at < 0.05

## Discussion

MRI findings of TMJ in TMD patients of different ages are still unclear. This study aimed to retrospectively analyze and compare the characteristics of MRI features of TMJs in different age groups. A total of 1760 TMJs from 880 patients were included in the study and divided into three groups: ≤ 18Y, 19-30Y and > 30Y. The results showed that except condylar morphology, there were significant differences in the distribution of disc morphology, disc position, joint effusion and joint motion among different age groups. And, there were significant differences in the distribution of the degree of anterior disc displacement, condyle morphology and joint effusion in different disc morphology among different age groups (except for the distribution of joint effusion in > 30Y), among which the first two types were correlated with the disc morphology, but not with the joint effusion.

MRI is considered as the gold standard for the diagnosis of TMD because it accurately reflects the state of soft tissue. DC/TMD combined with MRI can improve diagnosis specificity and sensitivity [18]. Clinically, MRI is often used to evaluate intra-articular soft tissue, like the shape and position of the articular disc, joint effusion. Dynamic MRI sequences are beneficial for the evaluation of morphology and function, especially in TMD patients [19]. In this study, in order to obtain the trend of MRI imaging features of the total sample size, TMJs on the left and right sides was compared, and no statistical difference was found. In terms of the shape of disc, biconcave ranked first followed by contracture, irregular and lengthened. And in terms of the position of disc, m-ADD ranked first followed by NA, s-ADD and PDD.

There are few studies on the morphology of articular disc at different ages, and the classifications various. Vervaeke et al. [5] divided it into crumpled, rounded, flat, and disc perforations. Vogl et al. [19] divided it into biconcave, biplane/flattened, degenerated. Fan et al. [20] divided it into oval shape, flat shape and beak-like shape. We divide the disc into biconcave, contracture, irregular and lengthened disc. The overall distribution trend of the shape of disc in 1760 TMJs is: biconcave ranked first followed by contracture, irregular and lengthened. There were significant differences in the distribution of articular disc morphology in different age groups. In ≤ 18Y group, the proportion of TMD patients with contracture discs ranked the first (37.2%), while in 19-30Y and > 30Y group, biconcave ranked first (33.7% and 36.4% respectively). The proportion of lengthened disc ranked the last in all the three age groups with about 14.4%.

Combined with clinical practice, we believe that morphology of articular disc may be related to the symptoms of TMD. Therefore, the correlation between articular disc morphology and articular disc position, joint effusion and condylar bone morphology at different ages was evaluated in this study.

The importance of articular disc position in TMD has been extensively studied [21, 22]. Among the 1760 TMJs, only 26% of discs with the posterior edge located in the normal 12 o'clock position with respect to the condyle, which smaller than luo et al. (33%) [23]. There were significant differences in the distribution of articular disc position in different age groups and the different articular disc morphology. The distribution of m-ADD ranked the first, accounting for about 61.8%. It should be noted that the proportion of PDD decreased significantly with the increase of age, and the proportion was greater than s-ADD in patients ≤ 18Y. We think it may be because the condyle is not fully developed in patients younger than 18 years of age [24]. Generally, the order of articular disc position in different articular disc morphology is as follows. 1) biconcave: normal ranked the first (60.7%), followed by s-ADD, m-ADD, PDD; 2) contracture: m-ADD ranked the first (96.4%), followed by normal, s-ADD, PDD; 3) lengthened: normal ranked the first, followed by m-ADD, s-ADD, PDD; 4) irregular: m-ADD ranked the first (97.0%), followed by normal, PDD and s-ADD (0.0%). However, in ≤ 18Y group, 72.4% of the positions of the biconcave disc were normal, followed by m-ADD, and m-ADD ranked first of lengthened disc. There was a correlation between the degree of anterior disc displacement and the disc shape in all age groups.

It has been reported that joint effusion primarily occur in joints with disc displacement and are strongly associated with joint pain [25, 26]. We found that there were statistically significant differences in the distribution of joint effusion among the three age groups. Of the 1760 TMJs, 83.3% were free of joint effusion and 91.2% were slight. The incidence of joint effusion of contracture disc was significantly higher than that of other types. However, There was no correlation between articular effusion and articular disc morphology.

We know that joint motion is related to disc displacement. Limited mouth opening may restricted by disc displacement, such as ADDWoR and ADDWR with intermittent locking [27–29]. Hypermobility may related with morphology of the musculoskeletal system[30]. There were significant differences in the distribution of joint motion among different age groups. The proportion of limited in ≤ 18Y group (54.4%) was significantly higher than that in 19-30Y group (29.5%) and > 30Y group (43.3%). It can be seen that most patients in the ≤ 18Y and > 30Y groups were accompanied by restricted mouth opening when visited the doctor.

Liu et al.[31] reported that MRI can detect condylar bone abnormalities, but could not efficiently detect the severity. There was no statistical difference in the distribution of condyle morphology in different age groups, but there was statistical difference and a correlation in the distribution of condyle morphology in different articular disc morphology. The condyle morphology was normal in 51.5% of 1760 TMJs. The morphology of articular disc that are easy to cause abnormal condylar bone are irregular, contracture, lengthened and biconcave in order. In > 30Y group, the proportion of condyle bone abnormality in contracture disc was 66.3%, which was significantly higher than that in other age groups.

## Conclusion

There were significant differences in MRI images of disc shape, disc position, joint effusion, joint motion and condyle morphology among different age groups. The morphology and position of the articular disc changed significantly with age, but the proportion of abnormal condylar bone remained about 50%. The greater the degree of disc folding, the more prone to bone abnormalities.

## Data Availability

The datasets generated and/or analysed during the current study are not publicly available due ethical concerns but are available from the corresponding author on reasonable request.

## Abbreviations

TMD: Temporomandibular disorder
TMJ: Temporomandibular joint
TMJ ID: TMJ internal derangement
NA: Normal
ADD: Anterior disc displacement
PDD: Posterior disc displacement
ADDWR: Anterior disc displacement with reduction
ADDWoR: Anterior disc displacement without reduction
CBCT: Cone beam computed tomography
MRI: Magnetic resonance imaging

## References

[1] Gonçalves DA, Dal Fabbro AL, Campos JA (2010). Symptoms of temporomandibular disorders in the population: an epidemiological study. J Orofac Pain.

[2] Schiffman E, Ohrbach R, Truelove E (2014). Diagnostic criteria for temporomandibular disorders (DC/TMD) for clinical and research applications: recommendations of the international RDC/tmd consortium network* and orofacial pain special interest groupdagger. J Oral Facial Pain Headache.

[3] Paesani D, Westesson PL, Hatala M (1992). Prevalence of temporomandibular joint internal derangement in patients with craniomandibular disorders. Am J Orthod Dentofacial Orthop.

[4] Talmaceanu D, Lenghel LM, Bolog N (2018). Imaging modalities for temporomandibular joint disorders: an update. Clujul Med.

[5] Vervaeke K, Verhelst PJ, Orhan K (2022). Correlation of MRI and arthroscopic findings with clinical outcome in temporomandibular joint disorders: a retrospective cohort study. Head Face Med..

[6] Tomas X, Pomes J, Berenguer J (2006). MR imaging of temporomandibular joint dysfunction: a pictorial review. Radiographics.

[7] Boboc AM, De Stefano A, Impellizzeri A (2022). Correlation between generalised joint hypermobility and temporomandibular joint disc displacement in adolescent patients: magnetic resonance imaging study. Eur J Paediatr Dent.

[8] Emshoff R, Innerhofer K, Rudisch A (2001). Relationship between temporomandibular joint pain and magnetic resonance imaging findings of internal derangement. Int J Oral Maxillofac Surg.

[9] Hosgor H (2019). The relationship between temporomandibular joint effusion and pain in patients with internal derangement. J Craniomaxillofac Surg..

[10] Luo D, Qiu C, Zhou R (2022). MRI-based observation of the size and morphology of temporomandibular joint articular disc and condyle in young asymptomatic adults. Dentomaxillofac Radiol.

[11] Demir MG (2022). Comparison of symptoms, signs, gender, and magnetic resonance images of temporomandibular joint disorder patients. Cranio..

[12] Gauer RL, Semidey MJ (2015). Diagnosis and treatment of temporomandibular disorders. Am Fam Physician.

[13] Orhan K, Nishiyama H, Tadashi S (2006). Comparison of altered signal intensity, position, and morphology of the TMJ disc in MR images corrected for variations in surface coil sensitivity. Oral Surg Oral Med Oral Pathol Oral Radiol Endod.

[14] Koh KJ, Park HN, Kim KA (2013). Relationship between anterior disc displacement with/without reduction and effusion in temporomandibular disorder patients using magnetic resonance imaging. Imaging Sci Dent.

[15] Litko-Rola M, Szkutnik J, Różyło-Kalinowska I (2021). The importance of multisection sagittal and coronal magnetic resonance imaging evaluation in the assessment of temporomandibular joint disc position. Clin Oral Investig.

[16] Jung YW, Park SH, On SW (2015). Correlation between clinical symptoms and magnetic resonance imaging findings in patients with temporomandibular joint internal derangement. J Korean Assoc Oral Maxillofac Surg.

[17] Jeon KJ, Kim YH, Ha EG (2022). Quantitative analysis of the mouth opening movement of temporomandibular joint disorder patients according to disc position using computer vision: a pilot study. Quant Imaging Med Surg.

[18] Shen S, Ye M, Wu M (2022). MRI and DC/TMD methods analyze the diagnostic accuracy of the change in articular disc of temporomandibular joint. Comput Math Methods Med..

[19] Vogl TJ, Günther D, Weigl P (2021). Diagnostic value of dynamic magnetic resonance imaging of temporomandibular joint dysfunction. Eur J Radiol Open..

[20] Fan WP, Liu MQ, Zhang XH (2019). MRI observation of condylar location and morphology in the patients with temporomandibular disc displacement. Zhonghua Kou Qiang Yi Xue Za Zhi.

[21] Tresoldi M, Dias R, Bracci A (2021). Magnetic resonance imaging evaluation of closed-mouth TMJ disc-condyle relationship in a population of patients seeking for temporomandibular disorders advice. Pain Res Manag.

[22] Zhao X, Xiong X, Sun W (2021). Symptoms, disc position, occluding pairs, and facial skeletal characteristics of older patients with temporomandibular disorders. J Int Med Res.

[23] Luo D, Yang Z, Qiu C (2022). A magnetic resonance imaging study on the temporomandibular joint disc-condyle relationship in young asymptomatic adults. Int J Oral Maxillofac Surg.

[24] Lei J, Liu MQ, Yap AU (2013). Condylar subchondral formation of cortical bone in adolescents and young adults. Br J Oral Maxillofac Surg.

[25] Westesson PL, Brooks SL (1992). Temporomandibular joint: relationship between MR evidence of effusion and the presence of pain and disk displacement. AJR Am J Roentgenol.

[26] Yano K, Sano T, Okano T (2004). A longitudinal study of magnetic resonance (MR) evidence of temporomandibular joint (TMJ) fluid in patients with TMJ disorders. Cranio.

[27] Heo HA, Yoon HJ (2020). Clinical outcomes of patients with bilateral anterior disc displacement without reduction and erosive change of the temporomandibular joint after performance of unilateral arthrocentesis and stabilisation splint therapy. J Oral Rehabil.

[28] Al-Baghdadi M, Durham J, Araujo-Soares V (2014). TMJ disc displacement without reduction management: a systematic review. J Dent Res.

[29] Takahara N, Imai H, Nakagawa S (2014). Temporomandibular joint intermittent closed lock: clinic and magnetic resonance imaging findings. Oral Surg Oral Med Oral Pathol Oral Radiol.

[30] Tuijt M, Parsa A, Koutris M (2018). Human jaw joint hypermobility: Diagnosis and biomechanical modelling. J Oral Rehabil.

[31] Liu SS, Xu LL, Lu SJ (2022). Diagnostic performance of magnetic resonance imaging for degenerative temporomandibular joint disease. J Oral Rehabil..

